# Ultrasound speckle tracking imaging measurement of endocardial longitudinal strain for evaluation of prognostic value of “new quadruple” therapy in patients with chronic heart failure

**DOI:** 10.3389/fendo.2025.1551927

**Published:** 2025-02-20

**Authors:** Man Tang, Yuwei Zeng, Ping Zhao, Qianlei Zhao

**Affiliations:** ^1^ Department of Ultrasound, The Affiliated People’s Hospital of Ningbo University, Ningbo, Zhejiang, China; ^2^ Department of Cardiology, The Affiliated People’s Hospital of Ningbo University, Ningbo, Zhejiang, China

**Keywords:** heart failure with reduced ejection fraction, two dimensional ultrasound speckle tracking, hierarchical strain imaging, new quadrupole, overall longitudinal strain of the left ventricle, subendocardial, major adverse cardiac events

## Abstract

**Purpose:**

This study aimed to evaluate the effectiveness of the “new quadruple” therapy in chronic heart failure (CHF) patients with metabolic syndrome using 2D speckle tracking imaging (2D-STI) stratified strain imaging to measure endocardial longitudinal strain while exploring its underlying neuroendocrine mechanisms.

**Patients and methods:**

The study retrospectively analyzed 158 patients with heart failure with reduced ejection fraction [HFrEF; left ventricular ejection fraction (LVEF) < 40%] treated with the “new quadruple” therapy (angiotensin receptor neprilysin inhibitor (ARNI), sacubitril/valsartan, dapagliflozin, bisoprolol, and spironolactone) for 8 weeks. Conventional ultrasound indices, left ventricular global longitudinal strain (LVGLS), and subendocardial longitudinal strain (LS) were measured pre- and post-treatment. Follow-up for 15 months recorded major adverse cardiac events (MACEs).

**Results:**

The 158 patients were divided into two groups: MACEs (n=25) and no MACEs (n=133). Univariate comparisons revealed significant differences between groups in coronary artery diameter stenosis percentage; admission LVEF and brain natriuretic peptide (BNP); LVGLS and subendocardial LS; post-treatment LVEF, LVGLS, and subendocardial LS, ΔLVGLS; and subendocardial ΔLS (P < 0.05). Multifactorial Cox regression modeling showed that coronary artery diameter stenosis, admission LVEF, BNP, subendocardial LS, post-treatment LVEF, and subendocardial LS were predictive factors for MACEs in HFrEF patients following “new quadruple” therapy (P < 0.05). ROC analysis indicates that post-treatment subendocardial LS predicts MACEs with an AUC of 0.871, which was significantly higher than other single metrics (P < 0.05).

**Conclusions:**

Using 2D-STI layer-specific strain imaging to measure endocardial longitudinal strain serves as a significant non-invasive indicator in predicting MACEs during 1-year follow-up after “new quadruple” therapy in HFrEF patients with metabolic syndrome, highlighting substantial clinical applicability. Additionally, our findings suggest that the therapy may improve prognosis through the modulation of neuroendocrine mechanisms.

## 1 Introduction

Heart failure poses a serious threat to human health. The number of heart failure patients worldwide is rising year by year, and the morbidity and mortality rates remain high, bringing a heavy burden to society and families. Exploring effective treatment methods for heart failure has become an urgent problem in the medical field. The “new quadruple” therapy has emerged in recent years, bringing new hope for heart failure treatment. The “new quadruple” therapy includes an angiotensin receptor neprilysin inhibitor (ARNI) or angiotensin-converting enzyme inhibitor (ACEI)/angiotensin II receptor antagonist (ACEI). It also includes angiotensin II receptor blockers (ARBs), beta receptor antagonists, Sali corticosteroid receptor antagonists, and a sodium-glucose cotransporter 2 inhibitor (SGLT2i) and is a therapy based on an in-depth study of the pathophysiologic mechanisms of heart failure. The therapy is designed to block the progression of heart failure at multiple points by targeting both hemodynamic abnormalities and neuroendocrine responses, which are key drivers of disease progression (1, 2). Neuroendocrine activation in heart failure involves the renin-angiotensin-aldosterone system (RAAS), sympathetic nervous system (SNS), and inflammatory pathways. These pathways contribute to myocardial stress, fibrosis, and fluid retention, which exacerbate the condition. The “new quadruple” therapy is aimed at addressing these mechanisms. The ARNI targets RAAS, beta blockers inhibit SNS activation, mineralocorticoid receptor antagonists (MRAs) prevent fluid retention, and SGLT2i potentially attenuates the cardiac metabolic stress caused by hyperglycemia and inflammation, all of which could have a profound impact on neuroendocrine regulation and disease progression (2, 3). Neuroendocrine dysregulation plays a critical role in the progression of heart failure and cardiac complications. These pathways contribute to myocardial stress, fibrosis, and adverse remodeling, increasing the risk of major adverse cardiac events (MACEs). Recent meta-analyses and clinical trials have demonstrated the efficacy of these therapies in improving patient outcomes, reducing mortality, and preventing hospitalizations (4, 5). Some studies emphasize the clinical relevance of layer-specific longitudinal strain measurements, with subendocardial strain emerging as a particularly sensitive biomarker for detecting early myocardial changes and predicting long-term outcomes in heart failure. The therapy has been validated in large randomized controlled trials and is recommended in clinical guidelines in several countries (6).

Despite these advances, the neuroendocrine mechanisms underlying heart failure progression and the effect of combined therapies like the “new quadruple” therapy on these mechanisms remain underexplored. While individual components of the therapy, such as ARNIs and SGLT2 inhibitors, have shown efficacy in reducing morbidity and mortality by improving hemodynamics and reducing neuroendocrine activation, their combined impact on the complex neuroendocrine feedback loop in heart failure patients has not been adequately addressed. This gap in knowledge warrants further investigation, especially in understanding how neuroendocrine responses to therapy can influence long-term outcomes (6, 7).

Heart failure prognosis remains a major concern for clinicians and patients, and factors that influence heart failure prognosis include the condition itself, pharmacological interventions, and patient execution, with cardiac structure and dysfunction likely to play a determining factor (3). Echocardiography is the tool of choice for evaluating the heart’s anatomy, and heart failure markers are the main indicators of the heart’s pumping function. With the continuous development of color Doppler ultrasound imaging, two-dimensional ultrasound spot tracking imaging (2D-STI) based on myocardial strain and stratified strain imaging are being increasingly used in clinical practice in hypertensive heart disease, myocardial infarction, cardiomyopathy, heart failure, and cardiotoxic injury after chemotherapy for malignant tumors and are closely related to disease prognosis (8–10).2D-STI is a non-invasive ultrasound technique used to assess myocardial strain by tracking the natural acoustic signals (speckles) in the myocardium. The method works by analyzing the movement of these speckles across multiple frames during the cardiac cycle, enabling the quantification of myocardial deformation. By measuring strain in various layers of the left ventricle, including the subendocardial, mid-myocardial, and subepicardial layers, 2D-STI provides detailed insights into regional myocardial function. This technique is particularly useful for detecting subtle changes in myocardial mechanics that are often overlooked by traditional imaging methods, making it an important tool in evaluating cardiac function in heart failure patients. As speckle tracking is sensitive to small myocardial strains, it can reflect the abnormalities of cardiac diastolic function at an early stage, among which left ventricular global longitudinal strain (LVGLS) is the most thoroughly studied and is recommended to be used as a routine screening index on admission to hospitals, which has important application value in evaluating the severity of the disease, the clinical efficacy, and the prognosis (11, 12). Given the irregular spherical structure of the heart and the three layers of myocardial anatomy with different orientations, layered strain can more objectively and accurately reflect abnormal local myocardial strain, which can provide valuable information for understanding the pattern of disease and more precise searching for lesions (13).

Heart failure remains a major concern due to its complex pathophysiology involving neuroendocrine dysregulation, myocardial dysfunction, and inflammation. While therapies such as ARNI, SGLT2 inhibitors, and MRAs have individually shown benefits in improving hemodynamics and reducing neurohormonal activation, their combined effects on the neuroendocrine mechanisms in heart failure have not been well explored. Previous studies have focused on the efficacy of individual therapies and biomarkers such as NT-proBNP in heart failure, but the impact of the “new quadruple” therapy on neuroendocrine responses and its association with disease progression remains unclear. Furthermore, while imaging techniques such as strain echocardiography are used to assess heart failure severity, few studies have examined how these imaging biomarkers correlate with the effects of combination therapies targeting neuroendocrine pathways. This gap in the literature highlights the need for a comprehensive study to evaluate the combined therapeutic effects of the “new quadruple” therapy on neuroendocrine responses and its impact on clinical outcomes in chronic heart failure patients (14).

Currently, there is a growing number of cases of chronic heart failure (CHF), particularly heart failure with reduced ejection fraction (HFrEF), undergoing “new quadruple” therapy in clinical practice in China. It is imperative to closely monitor patient prognoses. The aim of this study is to evaluate the effectiveness of the “new quadruple” therapy in CHF patients with metabolic syndrome, particularly its impact on neuroendocrine responses and cardiovascular outcomes using 2D-STI. Specifically, we seek to answer two key questions: (1) How does the “new quadruple” therapy affect neuroendocrine pathways in these patients? (2) Can 2D-STI serve as a reliable biomarker for predicting MACEs in patients receiving this therapy? We hypothesize that the combined therapy can modulate neuroendocrine pathways, including RAAS and SNS, and improve left ventricular strain measurements, ultimately reducing the risk of MACEs. Therefore, this study focuses on investigating the efficacy of 2D-STI layer-specific strain imaging in assessing the treatment outcomes and clinical prognosis of CHF patients undergoing “new quadruple” therapy, aiming to identify sensitive non-invasive indicators to guide clinical practice. This study addresses the gap in current research regarding the combined effect of these therapies on neuroendocrine dysregulation and myocardial strain in heart failure patients, particularly those with metabolic syndrome.

## 2 Material and methods

### 2.1 Subject information

This study retrospectively summarizes 158 patients with HFrEF diagnosed in our hospital from April 2022 to October 2023. This cohort included 89 men and 69 women, aged 42-79 years old, with an average age of 65.45 ± 5.90 years old. Inclusion criteria: 1) Meet the diagnostic criteria of HFrEF (8), admission left ventricular ejection fraction (LVEF) <40%; 2) receive the “new quadruple” therapy with no drug intolerance reaction; 3) 2D-STI stratified strain imaging images are clear, can be saved, and the measurement of parameters is accurate and reliable; 4) complete clinical and follow-up data. Exclusion criteria: 1) Acute heart failure, other types of chronic heart failure (e.g., ejection fraction preserved heart failure), and non-ischemic heart failure (e.g., heart failure caused by chemotherapeutic drugs, cirrhosis, congenital heart disease, cardiomyopathy, myocarditis, etc.); 2) patients change the drug regimen on their own in the middle of the day; 3) combining with other serious illnesses such as liver and renal dysfunction and malignant tumors. Our study conformed to the principles outlined in the Declaration of Helsinki (Br Med J 1964; ii: 177) and received ethical approval [Ningbo University Attached Human ethics review 2024 Research No.048] along with integral clinical and follow-up data.

### 2.2 Treatment

All the patients were admitted to the hospital for a complete examination and evaluation of the severity of the disease, and after obtaining the patient’s consent, the “new quadruple” therapy (**Table 1**) was recommended according to the guidelines (6).

**Table 1:** The “new quadruple” therapy regimen for heart failure treatment.

Medication	Brand name	Manufacturer	Dosage form	Starting dose	Target dose	Frequency	State drug license no.
Sacubitril/valsartan	Nosinotropic	Novartis Farma S.p.A., China	100mg × 14 tablets	50-100mg	200mg	Bis in Die (BID)	HJ20170363
Dapagliflozin	Andadan	AstraZeneca Pharmaceuticals	10mg × 14 tablets	10mg	10mg	Quaque Die (QD)	H20234463
Bisoprolol	Kang Xin	Merck Healthcare KGaA. Ltd.	20mg × 100 tablets	20mg	20mg	QD	H33020070

Course duration: 8 weeks.

### 2.3 Ultrasound

**Figure 1:**
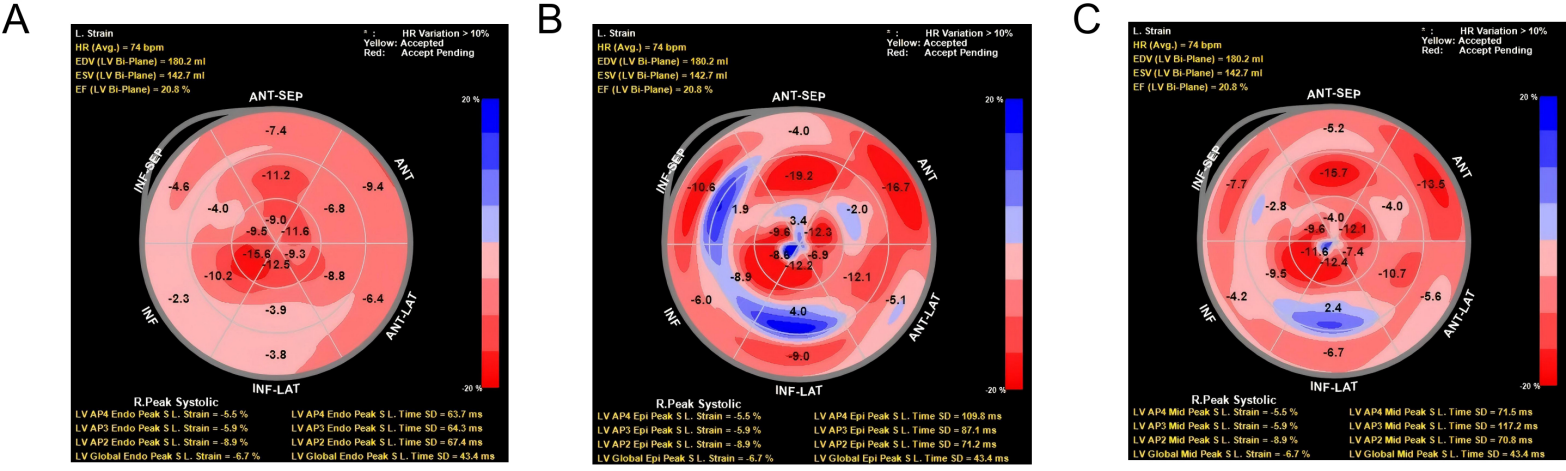
Two-dimensional longitudinal layered strain and strain curve of left ventricular myocardium. **(A)** Bull’s eye view of longitudinal strain in sub-endocardium. Radial strain data from the LV anterior-septal (ANT-SEP) and anterior (ANT) regions, showing peak systolic strain (LV APS End Peak S.L. Strain) and time to peak systolic strain (LV APS Peak S.L. Time) for each region. The strain values are represented with color intensity, where red indicates higher positive strain, and blue indicates negative strain. HR variation exceeding 10% is highlighted in yellow to red. **(B)** Bull’s eye view of longitudinal strain in sub-epicardium. Radial strain data from the LV right peak systolic region (R.Peak Systolic) and anterior-septal (ANT-SEP) and anterior (ANT) regions. The panel also displays strain measurements and time to peak systolic strain for the left ventricle, with color intensity indicating the magnitude of strain. Significant heart rate variation (HR >10%) is marked in red. **(C)** Bull’s eye view of longitudinal strain in midmyocardium. Radial strain data from the LV inferior-lateral (INF-LAT) region and anterior-septal (ANT-SEP) region. Strain measurements, including the peak systolic strain and time to peak systolic strain for the relevant regions, are depicted using a color scale. HR variation exceeding 10% is indicated in yellow to red, with corresponding LV strain parameters at the bottom.

The instrument is a Philips EPIQ 7C color Doppler ultrasound diagnostic instrument equipped with an X5-1 probe (frequency 1.0~5.0 MHz), automatic myocardial motion quantification (aCMQ) function, and Qlab image processing software (Version 10.5). The X5-1 probe was first used to routinely examine each section of the heart transthoracically to observe the morphology and structure of the heart, valvular activity, and hemodynamics, and several cardiac chambers are frozen. The left ventricular end-diastolic diameter and volume (LVEDD and LVEDV) are measured according to the biplane Simpson method and automatically calculated. The images are then imported into Qlab image processing software, and the stored dynamic cardiac chamber sections are sequentially selected and entered into the aCMQ mode of speckle tracking analysis. After clicking Draw, the tracing points were placed at the mitral annulus and apical endocardium on both sides of the mitral valve, and the software automatically tracked the myocardial motion to generate the region of interest. The edge of the endocardium and epicardium was adjusted manually so that it was in line with the thickness of the myocardium. Clicking Accept automatically generated the triple layer of the LV system. Clicking Accept again automatically generated strain values, strain curve diagrams, and bull’s-eye diagrams of the three layers of the myocardium (15, 16), and automatically calculated the left ventricular global longitudinal strain (LVGLS), and the subendocardial, mid-layer, and subepicardial LS (see **Figure 1**). The assessment was conducted by ultrasound specialists working in our hospital for at least 5 years. The image acquisition and parameter measurement were completed by a physician who has been working in our hospital for at least 5 years, and the consistency of each parameter was evaluated. The evaluation was expressed as the inter-observer and intra-observer intragroup correlation coefficients (ICC), and an ICC ≥ 0.75 is sufficient to determine good consistency of the parameters.

### 2.4 Observation indicators

General patient data were recorded, including sex, age, body mass index (BMI), underlying diseases (hypertension and diabetes), percentage of coronary artery diameter stenosis (as indicated by automatic measurements based on intraprocedural digital subtraction angiography and quantification software), serum brain natriuretic peptide (BNP) levels before treatment, and ultrasound indices including LVEDd, LVEDV, LVEF, LVGLS, subendocardial, mesial and subepicardial LS. After 8 weeks of treatment, LVEF, LVGLS, subendocardial, mesial, and subepicardial LS were measured and the difference was calculated as ΔLVEF = post-treatment - pre-treatment, ΔLVGLS improvement value(ΔLVGLS) = post-treatment - pre-treatment, and ΔLS in subendocardium = post-treatment-pre-treatment.

The routine follow-up cutoff was April 2024 and the duration of follow-up ranged from 9 to 24 months, with a median duration of 15.0 months. MACEs and time of occurrence were recorded; MACEs mainly included heart failure exacerbation, malignant arrhythmia, thrombosis, cardiogenic shock, and death.

### 2.5 Statistical methods

IBM SPSS 22.0 (IBM Corp., Armonk, NY, USA)) statistical software was used to express the measurement data conforming to a normal distribution as mean ± standard deviation, and the comparison between two groups was performed using an independent samples t-test. The measurement data not conforming to normal distribution was expressed as median and quartiles, and the comparison is performed using the Mann–Whitney U test, and the comparison of counting data [cases (%)] was performed using the χ^2^ test. The multifactorial Cox regression analysis was used to screen the risk factors for the occurrence of MACEs and the stepwise backward method was used. The working curve (ROC) of the participants was used to calculate the area under the curve (AUC), sensitivity, and specificity to obtain the optimal critical value, and the Z test was used to compare the AUCs. P < 0.05 was considered statistically significant.

## 3 Results

### 3.1 Comparison of clinical data and ultrasound indexes between the two groups of patients

The 158 patients were assigned to the MACEs group (15.82%, 25/158) and no-MACEs group (133). Univariate comparisons revealed significant differences in the percentage of coronary artery diameter stenosis; admission LVEF and BNP levels; pre-treatment LVGLS and subendocardial longitudinal strain (LS); post-treatment LVEF, LVGLS, and subendocardial LS; ΔLVGLS and subendocardial ΔLS; and duration of follow-up in the MACEs group compared with those of the no-MACEs group (P < 0.05, **Table 2**). Subendocardial LS was a more predictive metric for MACEs compared to other standard markers such as LVGLS and LVEF. This is due to the unique role of the subendocardial layer, which is more susceptible to ischemic injury and myocardial dysfunction in heart failure. It reflects early myocardial changes that are not yet detectable by traditional methods, providing a sensitive and localized measure of heart function. Our findings support subendocardial LS as a valuable non-invasive biomarker, offering superior predictive accuracy with a higher AUC for MACEs, making it a more reliable tool for early risk stratification in HFrEF patients.

**Table 2:** Comparison of clinical data and ultrasound indexes between the two groups of patients.

Events	NoMACEs group (n=133)	MACEs group (n=25)	Z/t/χ^2^ value	P-value
Follow-up time (months)	14.90±5.12	11.25±3.84	3.393	0.001
M/F	74/59	15/10	0.163	0.687
Age (years)	65.32±6.03	66.18±5.19	-0.669	0.505
BMI (kg/m^2^)	25.22±2.12	25.71±1.79	-1.092	0.276
Hypertension [cases (%)]	56 (42.11)	12 (48.00)	0.298	0.585
Diabetes mellitus [cases (%)]	30 (22.56)	7 (28.00)	0.348	0.555
Percentage of coronary artery diameter stenosis (%)	89.90±5.22	93.50±3.77	-3.290	0.001
Pre-treatment				
BNP (pg/mL)	1543.41±454.60	1804.66±378.61	-2.701	0.008
LVEF (%)	36.28±2.47	35.04±1.82	2.386	0.018
LVEDd (mm)	54.40±2.33	55.26±1.81	-1.743	0.083
LVEDV (mL)	183.07±29.91	187.53±24.87	-0.701	0.484
LVGLS (%)	-17.19±2.25	-15.83±2.24	-2.768	0.006
Subendocardial LS (%)	-19.07±1.73	-18.28±1.87	-2.081	0.039
Middle LS (%)	-16.99±1.40	-16.42±1.26	-1.882	0.062
Subepicardial LS (%)	-15.30±1.36	-14.93±1.31	-1.278	0.203
Post-treatment				
LVEF (%)	46.56±2.83	44.92±2.30	2.743	0.007
LVGLS (%)	-19.12±1.94	-16.61±2.17	-5.816	<0.001
Subendocardial LS (%)	-20.72±1.70	-18.16±1.64	-6.951	<0.001
⊿LVEF (%)	10.28±3.74	9.87±2.87	0.518	0.605
⊿LVGLS (%)	1.41 (0.76,2.67)	0.36 (-0.88,2.11)	-2.932	0.003
⊿Subendocardial LS (%)	1.39 (0.43,2.81)	-0.40 (-1.14,1.91)	-3.285	0.001

MACEs, major adverse cardiac events; BMI, body mass index; BNP, brain natriuretic peptide; LVEF, left ventricular ejection fraction; LVEDd, left ventricular end diastolic diameter and volume; LVEDV, left ventricular end-diastolic volume; LVGLS, left ventricular global longitudinal strain.

### 3.2 Risk factor analysis for MACEs

The above indicators with P < 0.05 (all continuous variables) were used as factors, and the prognosis for the occurrence of MACEs and time to occurrence were included in the multifactorial Cox regression model as outcome variables. The analysis shows that the percentage of coronary artery diameter stenosis, admission LVEF, BNP level, subendocardial LS, post-treatment LVEF, and subendocardial LS were predictive factors for MACEs in patients with HFrEF at 1 year of follow-up after the new quadruple treatment (P<0.05, **Table 3**).

**Table 3:** Risk factor analysis of MACEs in HFrEF patients at 1-year follow-up after “new quadruple” therapy.

Factor	β	SE	Wald	P-value	HR value	95% CI
Percentage of coronary artery diameter stenosis	0.133	0.050	7.014	0.008	1.142	1.035~1.260
BNP levels	0.002	0.001	10.593	0.001	1.002	1.001~1.004
Admission LVEF	-0.331	0.136	5.916	0.015	0.718	0.550~0.938
LVGLS before treatment	-0.186	0.180	1.062	0.303	0.831	0.584~1.182
Subendocardial LS before treatment	0.495	0.219	5.103	0.024	1.641	1.068~2.522
Post-treatment LVEF	-0.328	0.096	11.688	0.001	0.720	0.597~0.869
Post-treatment LVGLS	0.321	0.179	3.201	0.074	1.378	0.970~1.958
Subendocardial LS	0.597	0.173	11.969	0.001	1.817	1.296~2.549

### 3.3 Comparison of performance of predicted MACEs

**Figure 2:**
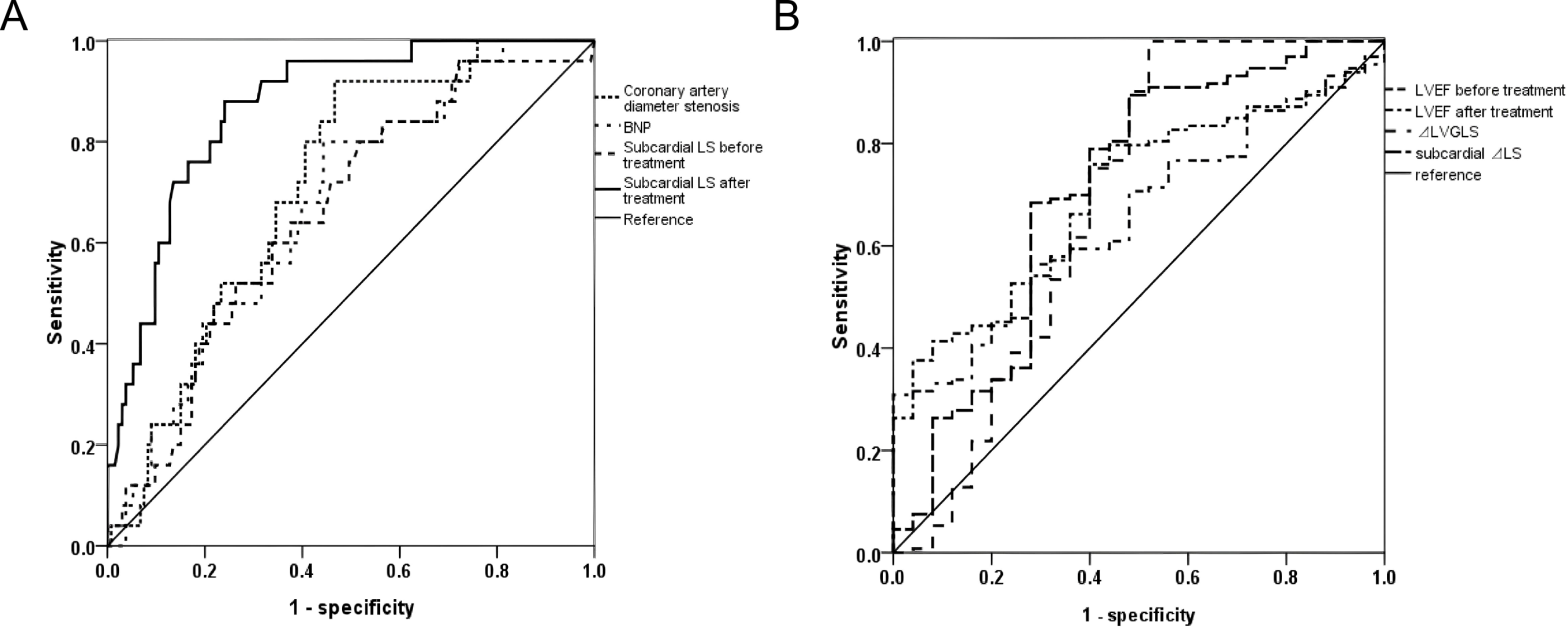
ROC curves for predicting MACEs in HFrEF patients at 1-year follow-up after “new quadruple” therapy. **(A)** ROC curve analysis comparing the diagnostic accuracy of various parameters, including coronary artery diameter stenosis, BNP, and subcardial LS before and after treatment. The sensitivity and specificity are plotted for each parameter, with the reference line indicating no discrimination between the conditions. The curves for each test are shown in dashed or solid lines, with varying levels of performance. **(B)** ROC curve analysis for the diagnostic accuracy of left ventricular ejection fraction (LVEF) before and after treatment, left ventricular global longitudinal strain (LVGLS), and subcardial LS. Each curve corresponds to a specific parameter, and the reference line represents the no-discrimination baseline. The curves help evaluate the ability of these parameters to differentiate between the clinical states.

The ROC showed that the AUC of subendocardial LS for predicting MACEs after treatment was 0.871, which was significantly higher than the other single metrics (P<0.05), with an optimal cutoff value of -19.05%, a sensitivity of 0.77, and a specificity of 0.83 (**Table 4**; **Figure 2**).

**Table 4:** Predicted performance of MACEs by metrics.

Norm	AUC	95% CI	P-value	Sensitivity	Specificity
Percentage of coronary artery diameter stenosis	0.715	0.622~0.808	0.001	0.64	0.65
BNP	0.676	0.572~0.780	0.005	0.68	0.67
Admission LVEF	0.656	0.556~0.756	0.014	0.70	0.62
Subendocardial LS before treatment	0.660	0.550~0.769	0.011	0.62	0.73
Post-treatment LVEF	0.700	0.604~0.797	0.001	0.65	0.66
Post-treatment subendocardial LS	0.871	0.805~0.936	<0.001	0.77	0.83
⊿LVGLS	0.685	0.538~0.832	0.003	0.71	0.58
Subendocardial LS	0.707	0.580~0.835	0.001	0.68	0.73

## 4 Discussion

In this study, the incidence of MACEs in HFrEF patients was 15.82% at 1-year follow-up after “new quadruple” therapy. Zheng et al. (7) registered a prospective observational multicenter cohort study (Trial Registration No. ChiCTR1800017204; 07/18/2018) for the prognostic assessment of heart failure stages in elderly hospitalized patients, with a total sample size of 1,068 individuals aged ≥65 years (mean age 75.3 ± 6.88 years), of whom 4.7% were healthy without risk factors for heart failure, 21.0% had stage A, 58.7% had stage B, and 15.6% had stage C/D; heart failure stage was associated with a worsening incidence of a MACE at 1 year (log χ^2^ = 69.62, P<0.001), and plasma NT-proBNP levels may help predict the risk of a MACE in stage B. This study showed that the percentage of coronary artery diameter stenosis; admission LVEF and BNP levels; LVGLS and subendocardial LS; post-treatment LVEF, LVGLS, and subendocardial LS; ΔLVGLS; ΔLVGLS; and subendocardial LS in the MACEs group differed significantly from those in the group without MACEs (P<0.05). Orru D’Ávila et al. (17), in a systematic review and meta-analysis, included 25 clinical studies (a total of 2,136 patients, of which 70.5% had heart failure with preserved ejection fraction) and showed that low LVGLS values were associated with low cardiorespiratory fitness (CRF) in patients with HFrEF, and that LVGLS may be a better predictor of CRF in patients with HFrEF compared to LVEF.

In this study, multifactorial Cox regression was used to show that percent stenosis of coronary artery diameter, admission LVEF, BNP, subendocardial LS, post-treatment LVEF, and subendocardial LS were predictive factors for MACEs at 1-year follow-up after “new quadruple” therapy in patients with HFrEF (P<0.05). Huttin et al. (18) conducted a 20-year follow-up study in the longitudinal familial STANISLAS cohort, which included 1,357 healthy subjects (51.6% female, aged 48.2 ± 14.1 years), and showed a high heritability of the subendocardial to subepicardial strain ratio (GLSEndo/GLSEpi), whereas the other classical parameters of left ventricular function were not. The GLSEndo/GLSEpi ratio is increasingly being recognized as an early and sensitive imaging biomarker of systolic dysfunction, and there may be an individual genetic susceptibility to reduced myocardial function (19, 20). Finally, the present study showed that the AUC of post-treatment subendocardial LS for predicting MACEs was significantly higher than that of other single indexes (P<0.05). The value of subendocardial LS of >-19.05% in HFrEF patients after the “new quadruple” therapy suggests that the risk of MACEs at 1 year of follow-up has better accuracy. It provides a simple and easy objective index for early screening of poor prognosis groups (21).

This study’s findings have significant implications for the stratified management and precision treatment of HFrEF. The integration of 2D-STI to assess layer-specific LS provides a powerful tool for early and precise risk stratification in HFrEF patients. By utilizing subendocardial LS as a non-invasive biomarker, this study presents a novel approach to monitoring disease progression and predicting MACEs, offering critical insights for tailoring individualized treatment strategies. These advancements contribute to the growing field of precision medicine by enabling clinicians to move beyond traditional metrics such as LVEF and LVGLS, instead focusing on more localized myocardial dysfunction that is better captured by layer-specific strain measurements. Moreover, the “new quadruple” therapy, by targeting multiple neuroendocrine pathways, exemplifies a shift towards a more comprehensive treatment approach in managing HFrEF. This multi-target therapy not only improves hemodynamics but also mitigates inflammation, fibrosis, and metabolic stress, crucial factors that contribute to the progression of heart failure. Our findings support the clinical applicability of this therapeutic regimen, especially when combined with advanced imaging techniques such as 2D-STI, in enhancing patient outcomes.

However, there are still some shortcomings in this study, such as limited sample size, single-center retrospective case summarization, relatively limited follow-up time, and a small number of positive cases, all of which could affect the stability of the results. Additionally, one potential limitation of our study is the influence of variations in patient adherence to the prescribed “new quadruple” therapy. Although adherence is not directly assessed in this retrospective analysis, it is known that medication adherence can significantly impact treatment outcomes in heart failure patients. Non-adherence to prescribed therapy may lead to suboptimal therapeutic effects, potentially confounding the observed results. Future studies could benefit from including a detailed assessment of adherence, such as medication refill records or patient self-reports, to better control for this factor. Additionally, patient education and monitoring during treatment could help mitigate the impact of adherence-related issues on the study outcomes.

While this study provides valuable insights, we acknowledge the limitations of the small sample size and the single-center design. These factors may limit the generalizability of our findings. To address these issues in future research, we recommend conducting multicenter studies with larger patient cohorts to improve statistical power and enhance the external validity of the results. Additionally, increasing the sample size could help identify more subtle effects and strengthen the conclusions drawn regarding the effectiveness of the “new quadruple” therapy. Moreover, prospective randomized controlled trials could further establish the causal relationships between neuroendocrine modulation, strain imaging parameters, and patient outcomes. This approach would provide a more robust evaluation of the therapy’s impact on HFrEF and help confirm the clinical applicability of subendocardial longitudinal strain as a predictive biomarker.

This study demonstrates the significant predictive value of subendocardial LS in identifying the risk of MACEs among HFrEF patients treated with “new quadruple” therapy. Subendocardial LS, with its superior sensitivity compared to LVEF and LVGLS, serves as a reliable biomarker for early risk stratification. The therapy’s multi-mechanistic approach, targeting RAAS suppression (ARNI) and metabolic stress reduction (SGLT2i), not only improves hemodynamics but also mitigates inflammation and fibrosis, thereby reducing MACEs and highlighting its transformative potential in heart failure management. These findings support the integration of advanced imaging techniques such as 2D-STI into routine practice and underscore the need for larger, multicenter studies to validate these results and refine personalized therapeutic strategies.

## 5 Conclusion

This study presents the innovative application of the guideline-recommended “new quadruple” therapy in treating patients with HFrEF. It emphasizes strict adherence to the regimen to obtain objective and stable 2D-STI stratified strain parameters. Among these, post-treatment subendocardial LS is a significant non-invasive indicator for predicting MACEs in HFrEF patients. Our findings suggest that subendocardial LS offers superior sensitivity to traditional indicators such as LVEF and LVGLS, positioning it as a valuable tool for early risk stratification. The study also underscores the multi-mechanistic benefits of the “new quadruple” therapy, which targets neuroendocrine pathways, reducing metabolic stress and inflammation, thereby improving hemodynamics and mitigating myocardial fibrosis. These effects collectively enhance cardiac function and reduce the risk of MACEs, presenting the therapy as a promising approach for HFrEF management. The study underscores the significance of 2D-STI and subendocardial LS in providing a more precise method for early risk stratification in HFrEF patients. This approach enhances the clinical management of heart failure by enabling more targeted and personalized treatment strategies, highlighting the importance of integrating advanced imaging techniques into routine clinical practice. The “new quadruple” therapy, with its multi-target approach, offers substantial clinical benefits by addressing neuroendocrine dysregulation, myocardial dysfunction, and metabolic stress, ultimately improving patient prognosis.

However, there are limitations to our study, including the small sample size, single-center retrospective nature, and relatively short follow-up period. These factors may impact the robustness of our findings, and the conclusions of this study need to be further validated through larger, multicenter prospective studies. Additionally, incorporating adherence assessments and further exploring the individual components of the therapy could provide deeper insights into its clinical efficacy.

In conclusion, our findings provide strong evidence for the utility of post-treatment subendocardial LS as a predictive biomarker for MACEs, highlighting its potential to refine the management of HFrEF. By incorporating advanced diagnostic tools, such as 2D-STI, into clinical practice, we can enhance personalized therapeutic strategies for heart failure patients. This study not only emphasizes the clinical utility of these techniques but also paves the way for future research to validate these results in larger, multicenter studies, ultimately improving the future management of HFrEF.

## Data Availability

The original contributions presented in the study are included in the article/supplementary material. Further inquiries can be directed to the corresponding author.

## References

[1] Alonso A Morris AA Naimi AI Alam AB Li L Subramanya V. Use of SGLT2i and ARNi in patients with atrial fibrillation and heart failure in 2021-2022: an analysis of real-world data. J Am Heart Assoc. (2024) 13:e032783. doi: 10.1161/JAHA.123.032783 38456406 PMC11010035

[2] Yan Y Liu B Du J Wang J Jing X Liu Y. SGLT2i versus ARNI in heart failure with reduced ejection fraction: a systematic review and meta-analysis. ESC Heart Fail. (2021) 8:2210–9. doi: 10.1002/ehf2.13313 PMC8120387 33749159

[3] Sharma A Verma S Bhatt DL Connelly KA Swiggum E Vaduganathan M. Optimizing foundational therapies in patients with HFrEF: how do we translate these findings into clinical care? JACC Basic Transl Sci. (2022) 7:504–17. doi: 10.1016/j.jacbts.2021.10.018 PMC9156437 35663626

[4] Omar AM Bansal M Sengupta PP. Advances in echocardiographic imaging in heart failure with reduced and preserved ejection fraction. Circ Res. (2016) 119:357–74. doi: 10.1161/CIRCRESAHA.116.309128 27390337

[5] Pozzi A Abete R Tavano E Kristensen SL Rea F Iorio A. Sacubitril/valsartan and arrhythmic burden in patients with heart failure and reduced ejection fraction: a systematic review and meta-analysis. Heart Fail Rev. (2023) 28:1395–403. doi: 10.1007/s10741-023-10326-1 37380925

[6] Tromp J Ouwerkerk W van Veldhuisen DJ Hillege HL Richards AM van der Meer P. A systematic review and network meta-analysis of pharmacological treatment of heart failure with reduced ejection fraction. JACC Heart Fail. (2022) 10:73–84. doi: 10.1016/j.jchf.2021.09.004 34895860

[7] Zheng PP Yao SM Guo D Cui LL Miao GB Dong W. Prevalence and prognostic value of heart failure stages: an elderly inpatient based cohort study. Front Med (Lausanne). (2021) 8:639453. doi: 10.3389/fmed.2021.639453 33968953 PMC8100028

[8] Zhang Y Xia L Mao Y Zhang X Xu J Huang J. Changes and significance of 2D-STI and right ventricular function parameters in evaluating cardiac function in patients with coronary heart disease and atrial fibrillation. Altern Ther Health Med. (2023) 29:40–4. 37235493

[9] Minciună IA Hilda Orășan O Minciună I Lazar AL Sitar-Tăut AV Oltean M. Assessment of subclinical diabetic cardiomyopathy by speckle-tracking imaging. Eur J Clin Invest. (2021) 51:e13475. doi: 10.1111/eci.13475 33326612

[10] Akbulut M Tan S Gerede Uludağ DM Kozluca V Dinçer İ. Evaluation of cardiac function in uncomplicated COVID-19 survivors by 2-dimensional speckle tracking imaging. Anatol J Cardiol. (2022) 26:841–8. doi: 10.5152/AnatolJCardiol.2022.1360 PMC9682558 35949116

[11] Murray J Bennett H Bezak E Perry R Boyle T. The effect of exercise on left ventricular global longitudinal strain. Eur J Appl Physiol. (2022) 122:1397–408. doi: 10.1007/s00421-022-04931-5 PMC9132819 35296909

[12] Yang Y Wu D Wang H Wang Y. Prognostic value of global longitudinal strain in hypertrophic cardiomyopathy: A systematic review and meta-analysis. Clin Cardiol. (2022) 45:1184–91. doi: 10.1002/clc.v45.12 PMC9748764 36177652

[13] Sivapathan S Geenty P Deshmukh T Boyd A Richards D Stewart G. Alterations in multi-layer strain in AL amyloidosis. Amyloid. (2022) 29:128–36. doi: 10.1080/13506129.2022.2026914 35188014

[14] Kjaer A Hesse B. Heart failure and neuroendocrine activation: diagnostic, prognostic and therapeutic perspectives. Clin Physiol. (2001) 21:661–72. doi: 10.1046/j.1365-2281.2001.00371.x 11722473

[15] Ananthapadmanabhan S Vo G Nguyen T Dimitri H Otton J. Direct comparison of multilayer left ventricular global longitudinal strain using CMR feature tracking and speckle tracking echocardiography. BMC Cardiovasc Disord. (2021) 21:107. doi: 10.1186/s12872-021-01916-8 33607946 PMC7893897

[16] Dobrovie M Bėzy S Ünlü S Chakraborty B Petrescu A Duchenne J. How does regional hypertrophy affect strain measurements with different speckle-tracking methods? J Am Soc Echocardiogr. (2019) 32:1444–50. doi: 10.1016/j.echo.2019.06.008 31377070

[17] Orru D'Ávila LB de Lima ACGB Milani M Milani JGPO Cipriano GFB Le Bihan DCS. Left ventricular global longitudinal strain and cardiorespiratory fitness in patients with heart failure: systematic review and meta-analysis. Hellenic J Cardiol. (2024) 79:58–69. 10.1016/j.hjc.2023.09.010 37778639

[18] Huttin O Xhaard C Dandine-Roulland C Le Floch E Bacq-Daian D Lamiral Z. Layer myocardial strain is the most heritable echocardiographic trait. Eur Heart J Cardiovasc Imaging. (2023) 24:1394–403. doi: 10.1093/ehjci/jead146 37352124

[19] Kitano T Nabeshima Y Abe Y Otsuji Y Takeuchi M. Accuracy and reliability of novel semi-automated two-dimensional layer specific speckle tracking software for quantifying left ventricular volumes and function. PLoS One. (2019) 14:e0221204. doi: 10.1371/journal.pone.0221204 31469858 PMC6716624

[20] Tan Y Wang L Hu B Chen C Jiang N Cao Q. Value of layer-specific speckle tracking echocardiography for early detection of myocardial injury caused by chemotherapy in breast cancer patients with cardiovascular risk. Int J Cardiovasc Imaging. (2022) 38:61–8. doi: 10.1007/s10554-021-02367-0 34363121

[21] Kornev M Caglayan HA Kudryavtsev AV Malyutina S Ryabikov A Schirmer H. Influence of hypertension on systolic and diastolic left ventricular function including segmental strain and strain rate. Echocardiography. (2023) 40:623–33. doi: 10.1111/echo.15625 37211961

